# New Journal

**Published:** 1874-01

**Authors:** 


New Journals.—Two new journals are shortly to be established. George M. Beard, M. D., of New York, announces that he intends early in the year to issue the first number of a semi-annual journal, *The Archives of Electrology and Neurology;* devoted to Electricity in its relations to Medicine and Diseases of the Nervous System. Drs. S. S. Jewell and Henry Bannister, of Chicago, also announce the proposed publication of a quarterly journal of Nervous and Mental Diseases

